# Da-Cheng-Qi Decoction Combined with Conventional Treatment for Treating Postsurgical Gastrointestinal Dysfunction

**DOI:** 10.1155/2017/1987396

**Published:** 2017-05-15

**Authors:** Wei Jin, Qingjie Li, Xiaoqiong Luo, Juan Zhong, Yang Song, Yiwei Li

**Affiliations:** ^1^Clinical Medicine College, Chengdu University of Traditional Chinese Medicine, Chengdu, Sichuan 610072, China; ^2^Emergency Department, The Teaching Hospital of Chengdu University of Traditional Chinese Medicine, Chengdu, Sichuan 610075, China; ^3^Department of Vascular Surgery, The Teaching Hospital of Chengdu University of Traditional Chinese Medicine, Chengdu, Sichuan 610075, China; ^4^Hearing Center/Hearing & Speech Science Laboratory, Department of Otorhinolaryngology-Head and Neck Surgery, West China Hospital of Sichuan University, Chengdu, Sichuan 610041, China

## Abstract

**Aim:**

To assess the current clinical evidence of the effectiveness of Da-Cheng-Qi Decoction (DCQD) for the treatment of Postoperative gastrointestinal dysfunction (PGD).

**Methods:**

Randomized controlled trails (RCTs) of Da-Cheng-Qi Decoction (DCQD) to PGD were searched from available major electronic databases to September 2016. The intervention must be a modified DCQD or DCQD integrated to Western Medicine (WM) compared with WM or placebo or blank. The main outcome index was clinical effectiveness and improvement of major symptoms. Data extraction, data analysis, and methodological quality assessment are conducted according to the Cochrane Handbook for Systematic Review of Interventions, version 5.0.2. RevMan 5.3 software was applied to our data analyses.

**Results:**

Seven RCTs involving 494 participants were recruited and identified. The methodological quality of all trials were assessed and generally of low-level. Those studies were published between 2004 and 2013. All 7 studies which used herbals (modified DCQD) integrate WM in test group compared with WM as the intervention and only one study (Sunyouxu 2013) integrates placebo to Western Medicine as the control group intervention. The treatment course was 1 week to 2 weeks. Evaluation of intervention effectiveness consists of the clinical effective rate indicator and the PGD symptoms indicator including time of borborygmus, time of gastrointestinal exhaust, and time of defecate. The clinical effectiveness results are beneficial to the test group.

**Conclusion:**

DCQD could improve PGD symptoms and promotion clinical effectiveness.

## 1. Introduction

Postsurgical gastrointestinal dysfunction (PGD) or postsurgical gastroparesis syndrome (PGS) is a serious generally nonmechanical obstruction complication of abdomens postoperative and it is a functional disorder characteristic as delayed gastric emptying [1–17]. It often happens after a few days or weeks or even years of surgery. It is said that the incidence rate of PGD/PGS is 1 : 1.6 ratio for male : female [1]. In a paper, 50–70% of postoperative pancreatic surgery patients suffer from this disease [18]. The pathogenesis is not clear but it is certain that stomach failure is not the cause and presentation [19]. In recent years, clinical practices often applied taken abrosia, gastrointestinal decompression, and choline drug resistance avoiding treating PGD/PGS but the efficacy was not guaranteed and remains of high recurrence rate [4], finding that alternative drugs to show efficacy for PGD/PGS are necessary.

In Chinese herbal medicine therapy, Da-Cheng-Qi Decoction (DCQD) is one of the most effective ways for PGD/PGS and it has long been used to treat PGD/PGS in clinical practice in China, which can significantly improve the symptoms and reduce the recurrence rate [20–23]. On the other hand, the lack of evidence based medical science makes this decoction unimpressive, so this systematic review aims to assess the current clinical evidence for the efficacy of DCQD for the treatment of PGD by conducting literature reviews in databases for randomized controlled trails (RCTs).

## 2. Materials and Methods

### 2.1. Databases and Searches

We searched all related trails or studies online and the databases we searched are as follows: PubMed, Embase, Cochrane library, CNKI, (Chinese Scientific Journal Database) VIP, Chinese Biomedical Literature Database (CBM), Medline, Chinese National Knowledge Infrastructure, and Wanfang database. The search strategies we used are as follows:

  #1 Traditional Chinese Medicine
  #2 Dachengqi Decoction
  #3 Integrated Chinese and Western medicines
  #4 #1–#3/OR
  #5 Gastroparesis
  #6 Gastrointestinal dysfunction
  #7 postsurgical gastrointestinal dysfunction
  #8 #5–#7/OR
  #9 #4 AND #8
  Searching Other Resources

In order to find out additional trails, we scanned references listed at the end of the identified publications, and we connected authors by email or telephone for data, if necessary. References of some other related published systematic reviews also are retrieved from the electronic databases.

All studies that we searched were published prior to September 11, 2016.

### 2.2. Study Selection

All RCTs are restricted to that DCQD alone as the intervention or western medicine assistant intervention in test group compared with the control group. We assessed all forms of DCQD, containing the modified DCQD, suppository drug of DCQD, or other forms of DCQD, whereas the control interventions prefer western medicinal therapy, placebo, and blank (no treatment) rather than any form of Traditional Chinese Medicine, such as acupuncture, moxibustion therapy, and massage that evaluated patients who were selected in spite of the nationality, age, and gender, but the patients who belong to any kind of postoperative gastroparesis syndrome patients scope (postcholecystectomy) were the only option. So the gastroparesis caused by diabetes patients was excluded. The main outcome was clinical effectiveness, which was assessed according to the improvement of gastrointestinal (GI) symptoms, and adverse events were assessed also. Duplicated publications were ascertained based on the reporting of the same patients and leaving only one paper as the usable literature.

### 2.3. Data Extraction and Quality Assessment

The literature researched, study selection, and data extraction are carried out by two group members dependently and selectively (literature, study selection, and study extraction). The extraction of studies included the study ID, the sample size, the gender, the specified method in experimental group and the control group, the course of treatment, main outcome measures, and the baseline report. If conflict, a mechanism for negotiated solution was set up and the divergence would be solved based on this mechanism (through discussion) and ask for additional information from a third authoritative party unless necessary. Two authors assessed the methodological quality of all included trails independently and based on the items or details noted in the Cochrane Handbook for Systematic Review of Interventions, Version 5.0.2 [13]. The items or details are as follows:

1. Sequence generation (selection bias).
2. Allocation concealment (selection bias).
3. Blinding of participants; personnel (performance bias).
4. Blinding of outcome assessors (detection bias).
5. Incomplete outcome data (attrition bias).
6. Selective outcome reporting (reporting bias).
7. Other sources of bias (other biases).

We judged each item on three levels (“Yes” is for low risk bias and presented that all the details were reduced to “Yes,” “No” is for a high risk of bias and presented that at least one detail was “No,” and “Unclear” means “unclear risk of bias” and intended that at least one item was “Unclear”) according the handbook.

### 2.4. Statistical Analysis

RevMan 5.3 software was applied to data analyses, in which all usable data were pooled and analyzed, dichotomous data were expressed as odd relative (OR), and continuous outcomes were expressed as mean difference (MD), with 95% CI. In the process of data analysis, if the heterogeneity was smaller than 75% (*I*^2^ < 75%), we applied the fixed effect model, and while the heterogeneity was larger than 75% (*I*^2^ < 75%), we used the random effect model.

## 3. Results

### 3.1. Description of Studies

**Figure 1:**
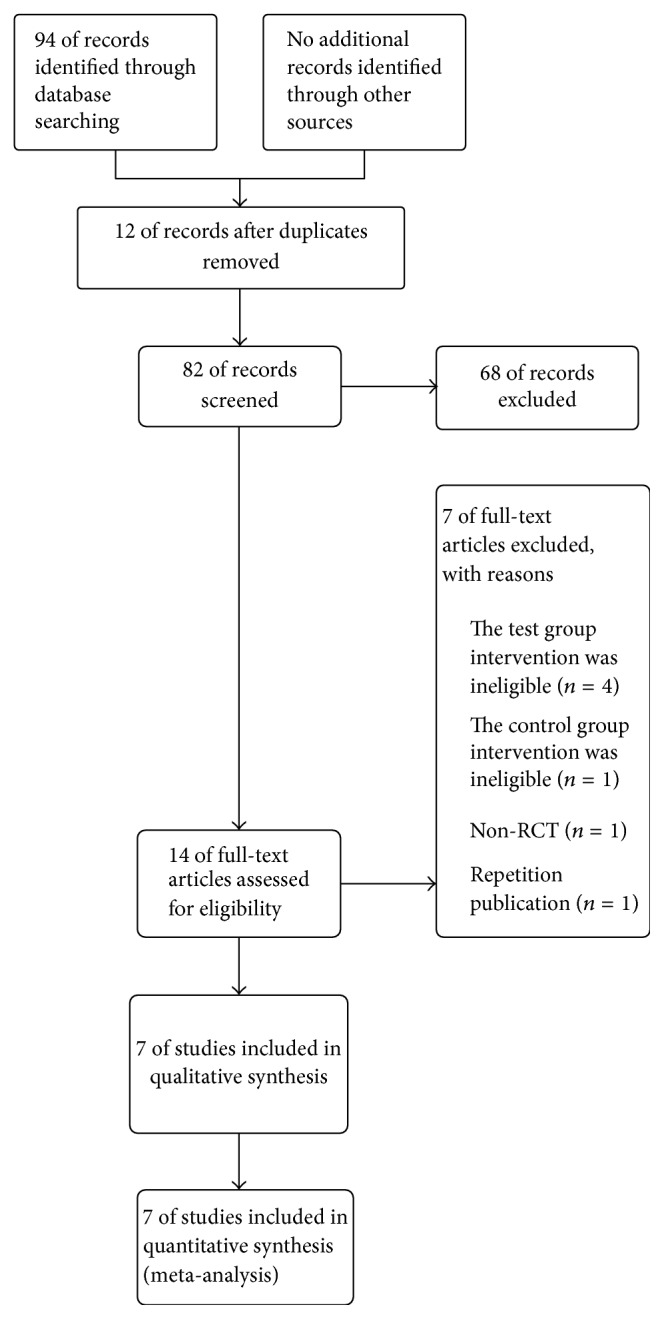
The flow chart of the literature screening.

There are 94 papers that were sought out from the electronic databases in total at the first step. Twelve papers were removed due to duplicates. Then, we passed by 68 papers through screening the title and the abstract due to the non-RCT but case reports, clinical observations, animal experimental studies, mechanism research studies, repetition papers, diabetic gastroparesis, and literature reviews. After full-text read to the remaining, three were excluded due to Xiao-Cheng-Qi Decoction [24–26] as the intervention measure, which is similar to Da-Cheng-Qi Decoction. One study was excluded due to the fact that intervention is Da-Cheng-Qi Integrated Massage [27]; another one was abandoned due to the fact that intervention is western medicine integrated normal saline (NS) [28] in control group; the third one was passed because of a nonrandomized trial [29]; and the last one was excluded due to the repetition publication [30]. As a result, 7 studies [31–37] (one English paper and 6 Chinese papers) involving 494 patients meet our inclusion criteria. Those studies were published between 2004 and 2013. The flow chart of the literature screening was present in Figure 1.

Among the 7 studies [31–37], all patients pertain to the postoperative gastroparesis patients and the experimental interventions were two forms: one anal suppository trial (Sunyouxu 2013) and six oral administration trails (Shen 2004, Qi 2007, Huodongmei 2008, Qiu 2009, Chenxiaoke 2011, and Huang 2012). Six trials contained 221 males and 124 female subjects with age ranging from 18 to 65 years and the other one (Huodongmei 2008) has not reported the number of men and women. All 7 studies which used herbals (modified DCQD) integrate the western medicine in control group as the intervention and only one study (Sunyouxu 2013) integrates placebo to western medicine as the control group intervention may be in order to touch a better randomized control effect and achieve stable test compliance into two groups. In another 6 studies, 3 control groups of western medical intervention were not specified and 3 were Mosapride (Chenxiaoke 2011), Magnesium Isoglycyrrhizinate (Huang 2012), and Metoclopramide (Shen 2004), respectively. The treatment course was 1 week to 2 weeks. Outcome measures were clinical effective rate, GI symptoms, and adverse events. Characteristics of all included studies are shown in Table S1 in Supplementary Material available online at https://doi.org/10.1155/2017/1987396.

### 3.2. Methodological Quality of Included Trials

We summarized the quality of all included trials and graph in forms of picture applied RevMan5.3 software. The sample size of all included trials ranged from 52 to 103 patients. Seven trails that we all pooled have not reported the details of sample size calculation. One trail written by Sunyouxu in year of 2013 is a double-blind placebo controlled trial but the used allocation concealment method and the blinding procedures were absent, while other papers have not mentioned the contents related to allocation concealment and blinding. One trail carried out by Qiu and his partner in year of 2009 described that they applied random number tables to reach the randomization, whereas the remaining 6 studies (Sunyouxu 2013, Qi 2007, Huodongmei 2008, Shen 2004, Chenxiaoke 2011, and Huang 2012) just simply mentioned “random allocation” of all patients into 2 groups. Six papers described the characteristics of all included patients (Sunyouxu 2013, Shen 2004, Qi 2007, Qiu 2009, and Chenxiaoke 2011), and all papers reported the baseline similarities between test group and control group. One study (Sunyouxu 2013) reported no participants' withdrawal and the remaining 6 studies have not noted the dropout of any participants, which bring us some difficulties to ascertain whether those studies are with an attrition bias or not. Two studies (Sunyouxu 2013 and Chenxiaoke 2011) reported the adverse events, but none reported follow-up of all participants.

### 3.3. Effect of the Interventions

The effect of the interventions was divided into 2 parts based on the clinical effective rate indicator and the GI symptoms indicator.

**Figure 2:**
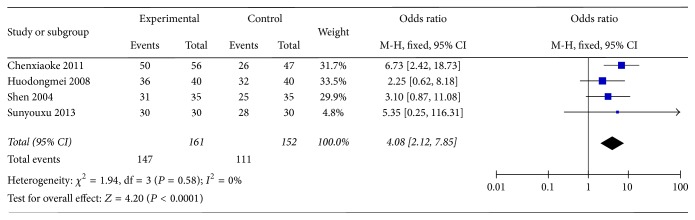
The clinical effective rate of DCQD.

Four studies (Sunyouxu 2013, Shen 2014, Huodongmei 2008, and Chenxiaoke 2011) presented the GI-fundamental clinical effective rate after being intervened for period of time, which is considered to be the main outcome measure. We pooled this 4 studies and the results are beneficial to the test group (*n* = 313, OR = 4.08, 95% CI: 2.12–7.85, *Z* = 4.20, and *P* < 0.0001) (Figure 2).

**Figure 3:**
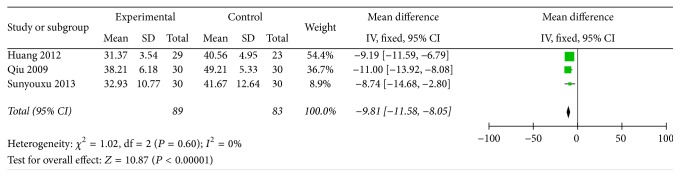
Improvement of time of borborygmus.

**Figure 4:**
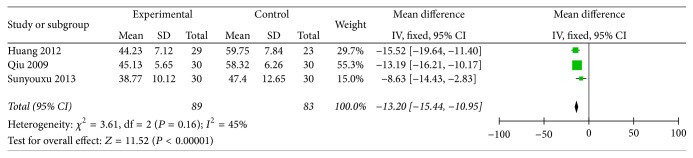
Improvement of time of gastrointestinal exhaust.

**Figure 5:**
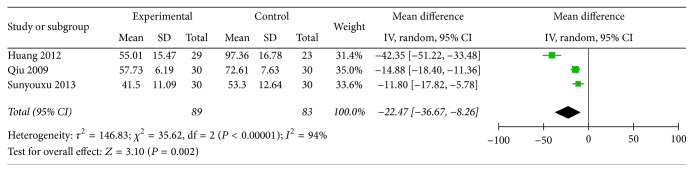
Improvement of time of defecation.

GI symptoms indicator was divided into 3 items: time of borborygmus, time of gastrointestinal exhaust, and time of defecation; those times were evaluated with hour. There are three papers with the description of three-item measurement. In the three papers, those indicators were showed with quantitative data and the mean standard deviation was used to data analysis. In condition of experimental group compared with control group, when *P* value of time variations was less than 0.05, the difference was statistically significant. We pooled these data and found that all items are beneficial to test group, and time of borborygmus was (*n* = 172, OR = −9.81, 95% CI: −11.58, −8.05, *Z* = 10.87, and *P* < 0.00001) (Figure 3) and time of gastrointestinal exhaust was (*n* = 172, OR = −13.2, 95% CI: −15.44, −10.95, *Z* = 11.52, and *P* < 0.00001) (Figure 4). Time of defecate item was pooled and exhibited heterogeneity (*I*^2^ = 94). Thus, the Random-effects model is used to statistically analyze, and the test group scored significantly higher than the control group (*n* = 172, OR = −22.47, 95% CI: −36.67, −8.26, *Z* = 3.1, and *P* = 0.002) (Figure 5), so the clinical effective rates of those indicators are unknown.

### 3.4. Risk of Bias

**Figure 6:**
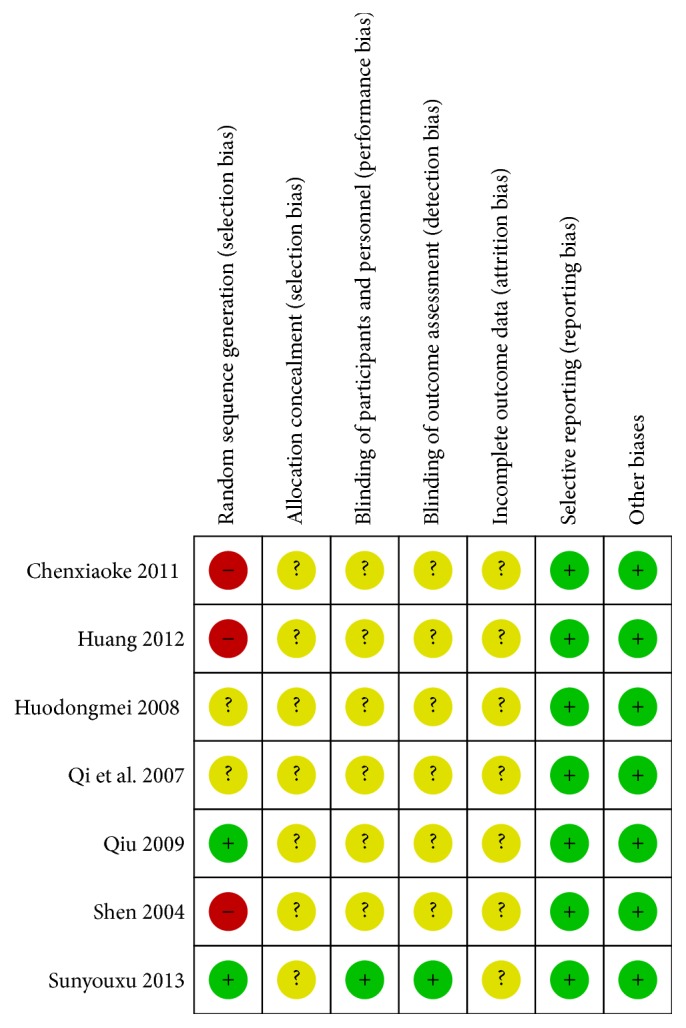
Risk of bias summary: review authors' judgements about each risk of bias item for each included study.

**Figure 7:**
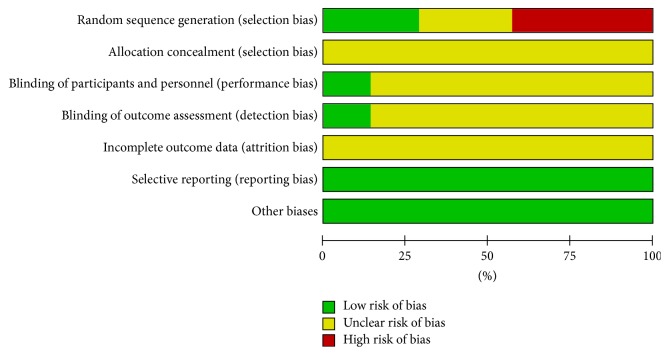
Risk of bias graph: review authors' judgements about each risk of bias item presented as percentages across all included studies.

Risk of bias of all included studies was based on the Cochrane Collaboration's tool, version 5.0.2. Risk of bias summary and graph are shown in Figures 6 and 7, respectively. And the details are as follows.

#### 3.4.1. Random Sequence Generation (Selection Bias)

A paper written by Sunyouxu in 2013 and another paper written by Qiu in 2009 applied the table of random number method as the randomization method, and those random sequence generation selection biases were low. Paper by Huodongmei 2008 and Qi 2007 just mentioned “random allocation” and no details were provided; therefore, the random sequence generation selection biases were unclear. The remaining studies by Shen 2004, Chenxiaoke 2011, and Huang 2012 remain unclear and the random sequence generation selection biases were high.

#### 3.4.2. Allocation Concealment (Selection Bias)

Seven included studies with no details on allocation concealment and the allocation concealment selection bias were all unclear.

#### 3.4.3. Blinding of Participants and Personnel (Performance Bias) and Blinding of Outcome Assessment (Detection Bias)

In our included studies, only Sunyouxu, 2013, mentioned blinding to all participants and assessors and others with no details offered; therefore the performance bias and detection bias were unclear but for study by Sunyouxu 2013 were low.

#### 3.4.4. Incomplete Outcome Data (Attrition Bias)

All studies of incomplete outcome date remain unclear due to insufficient reporting of attrition/exclusions in all studies and to permit judgement of “Yes” or “No.” There are no missing outcome data reported and no reasons for missing data provided in all studies.

#### 3.4.5. Selective Reporting (Reporting Bias)

All studies protocol is not available but it is clear that the published reports include all expected outcomes, and no reporting biases are shown.

#### 3.4.6. Other Biases

All studies appear to be free of other sources of bias and no potential of other biases.

### 3.5. Adverse Events

Two studies reported the adverse events (Sunyouxu 2013 and Chenxiaoke 2011). In the paper by Chenxiaoke 2011, light diarrhea is the major side effect into 2 groups, 1 in control group and 6 in test group, respectively. But all light diarrhea patients can continue to take pills and no measures were taken to solve this problem. In another paper (Sunyouxu 2013), no adverse events happened and were reported.

### 3.6. Follow-Up

Seven studies lack the description of follow-up participants.

## 4. Discussion

The functional gastrointestinal disorders of postoperative of PGD may lead to the digestive system not working for a few days, abdominal dissension, constipation, and so on. It is caused by intraoperative wound traction or lack of visceral perfusion which have been considered as one of the most common complications of abdominal surgeries under general anesthesia. In year of 2009, incidence of gastrointestinal dysfunction induced by opioid drugs was as high as 81% in the United States [38]. Prevalence of PGD is difficult to estimate due to the lack of accurate reporting in China. Clinically, most of the patients are disturbed by an obvious uncomfortable syndrome caused by PGD complicate symptoms. However, current management countermeasures to those disorders are far from clinical satisfactory [39]. In recent years, Chinese herbal medicine, especially Xiang-Sha-Liu-Jun-Zi Decoction and Cheng-Qi series of decoctions are widely used to manage PGD and timely flatus and defecation after abdominal operation suggests that Chinese herbal medicine is feasible method to PGD [31–37]. Our study aims to assess the current clinical evidence of the effectiveness of Da-Cheng-Qi Decoction for the treatment of PGD.

No Da-Cheng-Qi Decoction for the treatment of PGD systematic reviews was reported before us. Our review included 7 randomized trails and a total of 494 participants. The main findings of this paper were that DCQD demonstrated potential effects on the improvement of the PGD clinical effect (*n* = 313, OR = 4.08, 95% CI: 2.12–7.85, *Z* = 4.20, and *P* < 0.0001) compared with the control group. However, the relative low-level methodological quality of those trails may influence the result to some extent.

None of studies reported sample size calculations; therefore, the without planning sample size would make the results not be ascertained. Thus, the reliability of the outcome might be questionable. Two trails (Sunyouxu 2013 and Qiu 2009) make description of the randomization methods and others just mentioned “random allocating.” Only paper by Sunyouxu 2013 reported the allocation concealment, which would include false “RCTs” and furtherly mislead the results. Just paper by Sunyouxu 2013 demonstrated the method for blinding, which made it difficult to distinguish the performance and detection bias from the result and the lack of description.

PGD is a series of syndromes and some questionnaire/index such as the Chinese version of the Gastrointestinal Quality of Life Index or the Gastroparesis Cardinal Symptom Index should be used to estimate the effect if possible. We cannot find a universal standard/guideline/scale to guide the clinical effect assessment and we suggest that more feasible scale/clinical effect assessment methods would be promoted.

Only two studies which reported the adverse events (Sunyouxu 2013 and Chenxiaoke 2011) refer to the fact that no adverse events happed and to light diarrhea, respectively. Drug Safety is a serious issue that should be noted in detail, but most of included studies did not describe the adverse events of DCQD. No trials tell us the case of the patients' withdrawal, which could mislead us to make mistakes in the judgement of attribution bias of trials. The postoperative recovery of a patient is a long-term and necessary issue, but no studies made and noted the follow-up in all of 7 studies.

All in all, the DCQD can improve symptoms of PGD and is beneficial to functional gastrointestinal disorders patients compared with the control group which could be thought of as an alternative method for the treatment of PGD but our conclusion should be read with caution due to the poor methodological quality. In the future, well-designed RCTs and complete efficacy assessment system of CHM for PGD are urgently needed.

## Supplementary Material

Table S1: Characteristics of all included studies.
