# Supplementary material for: Da-Cheng-Qi Decoction Combined with Conventional Treatment for Treating Postsurgical Gastrointestinal Dysfunction
Source: Evid Based Complement Alternat Med. 2017 May 15;2017:1987396. doi: 10.1155/2017/1987396 (PMC5592004; doi:10.1155/2017/1987396)
Supplement: Supplementary file 1 — Table S1: Characteristics of all included studies. [file 1987396.f1.docx]

Table S1: Characteristics of all included studies.

| Trials | sample size | gender(E/C) | Experimental group | Control group | Period | Outcome measure | Balance report of baseline |
| --- | --- | --- | --- | --- | --- | --- | --- |
| Sun2013[31] | 60(30/30) | (14M:16F)/(15M:15F) | Western medicine + Modified Da cheng qi suppository,qd | Western medicine+placebo | 1 week | Clinical effective rate GI symptoms Adverse events | P > 0.05 |
| Shen2004[32] | 70(35/35) | 32M/38F | Metoclopramide + Modified Da cheng qi decoction,bid | Metoclopramide,20mg,bid | 1 week | Clinical effective rate | No significant differences |
| Qing2007[33] | 69(34/35) | (13M:21F)/(14M:21F) | Western medicine + Modified Da cheng qi decoction,bid | Western medicine,bid | 1 week | EGG,GI symptoms,Plasma motilin | P > 0.05 |
| Huo2008[34] | 80(40/40) | Not mentioned | Western medicine + Modified Da cheng qi decoction,bid | Western medicine,bid | 1 week | Clinical effective rate | No significant differences |
| Qiu2009[35] | 60(30/30) | (18M:12F)/(21M:9F) | Western medicine + Modified Da cheng qi decoction,bid | Western medicine,bid | 1 week | GI symptoms | P > 0.05 |
| Chen2011[36] | 103(47/56) | 66M/82F | Mosapride + Modified Da cheng qi decoction,bid | Mosapride,5mg,tid | 14 days | Clinical effective rate Adverse events | P > 0.05 |
| Huang2012[37] | 52(29/23) | (15M:14F)/(13M:10F) | Magnesium isoglycyrrhizinate + Modified Da cheng qi decoction,bid | Magnesium isoglycyrrhizinate,150mg,qd | 1 week | GI symptoms | P > 0.05 |
